# Antimicrobial peptide 2K4L inhibits the inflammatory response in macrophages and *Caenorhabditis elegans* and protects against LPS-induced septic shock in mice

**DOI:** 10.1038/s41598-024-64511-9

**Published:** 2024-07-02

**Authors:** Fangyu Ji, Guoxu Tian, Dejing Shang

**Affiliations:** 1 School of Life Science, Liaoning Normal University, Dalian, 116081 China; 2 Liaoning Provincial Key Laboratory of Biotechnology and Drug Discovery, Liaoning Normal University, Dalian, 116081 China

**Keywords:** Peptide, *C. elegans*, *A. baumannii*, LPS, Inflammation, Peptides, Cell biology

## Abstract

2K4L is a rationally designed analog of the short α-helical peptide temporin-1CEc, a natural peptide isolated and purified from the skin secretions of the Chinese brown frog *Rana chensinensis* by substituting amino acid residues. 2K4L displayed improved and broad-spectrum antibacterial activity than temporin-1CEc in vitro. Here, the antibacterial and anti-inflammatory activities of 2K4L in macrophages, *C. elegans* and mice were investigated. The results demonstrated that 2K4L could enter THP-1 cells to kill a multidrug-resistant *Acinetobacter baumannii* strain (MRAB 0227) and a sensitive *A. baumannii* strain (AB 22933), as well as reduce proinflammatory responses induced by MRAB 0227 by inhibiting NF-κB signaling pathway. Similarly, 2K4L exhibited strong bactericidal activity against *A. baumannii* uptake into *C. elegans*, extending the lifespan and healthspan of the nematodes. Meanwhile, 2K4L alleviated the oxidative stress response by inhibiting the expression of core genes in the p38 MAPK/PMK-1 signaling pathway and downregulating the phosphorylation level of p38, thereby protecting the nematodes from damage by *A. baumannii*. Finally, in an LPS-induced septic model, 2K4L enhanced the survival of septic mice and decreased the production of proinflammatory cytokines by inhibiting the signaling protein expression of the MAPK and NF-κB signaling pathways and protecting LPS-induced septic mice from a lethal inflammatory response. In conclusion, 2K4L ameliorated LPS-induced inflammation both in vitro and in vivo.

## Introduction

Inflammation is an immune response that exerts a protective effect on the balance of endogenesis when stimulated by pathogens, oxidative stress, or exposure to endotoxins such as lipopolysaccharide (LPS)^1,2^. Excessive inflammation can produce a series of inflammation-related diseases, such as endotoxemia and septic shock. Septic shock is an overwhelming microbe-induced inflammatory response that leads to organ dysfunction. It is a major public health problem due to high mortality rates and lack of treatment options^3^. Pathogen-induced inflammation results in immune system evasion, sustained inflammation and increased antimicrobial resistance^4^. *Acinetobacter baumannii* is one of the most multidrug-resistant pathogens of the ESKAPE group (*Enterococcus faecium*, *Staphylococcus aureus*, *Klebsiella pneumoniae*, *Acinetobacter baumannii,* *Pseudomonas aeruginosa* and *Enterobacter spp.*), which causes hospital-acquired infections leading to excessive inflammatory reactions, sepsis, septic shock and even death^5^. Recently, *A. baumannii* has become increasingly important because it has extensive drug resistance to all clinical antibiotics, and the *A. baumannii* infection mortality rate can reach 35%. Higher mortality rates were found in patients with drug-resistant strains compared to those with drug-susceptible strains due to the greater severity of illness and inappropriate empirical antibiotic treatment. Finding an effective method to control septic shock against the ESKAPE group has become an important target of clinical biologists.

Antimicrobial peptides (AMPs) act on defense systems and have relatively potent and short-lived effects against many microorganisms, including multidrug-resistant (MDR) pathogens. Unlike antibiotics with a single bacterial target, AMPs are able to target the bacterial membrane system by electrostatic interactions and damage membrane integrity, leading to the leakage of cytoplasmic components and microbial death^6^, which are expected to solve the current clinical problems.

The antimicrobial peptide 2K4L and 2K2L are the analog of temporin-1CEc, a natural peptide from the skin secretion of *Rana chensinensis*^7^. 2K4L and 2K2L are with 13 amino residues and 5 positive charges, but with different mean hydrophobicities of 17.14 and 14.94, respectively. Our previous study demonstrated that 2K4L exhibited broad-spectrum antibacterial activity than 2K2L, especially against the most multidrug-resistant pathogens of the ESKAPE group. In the present study, the antibacterial and anti-inflammatory activity of 2K4L in human macrophages and *C. elegans* exposed to *A. baumannii* was investigated. In addition, the anti-inflammatory mechanisms of 2K4L in *A. baumannii*-exposed THP-1 cells and *C. elegans* were explored. Furthermore, the protective action of 2K4L was elucidated in an LPS-induced septic shock mouse model.

## Results

### 2K4L kills intracellular MRAB 0227 and inhibits the inflammatory response in infected THP-1 cells

**Figure 1:**
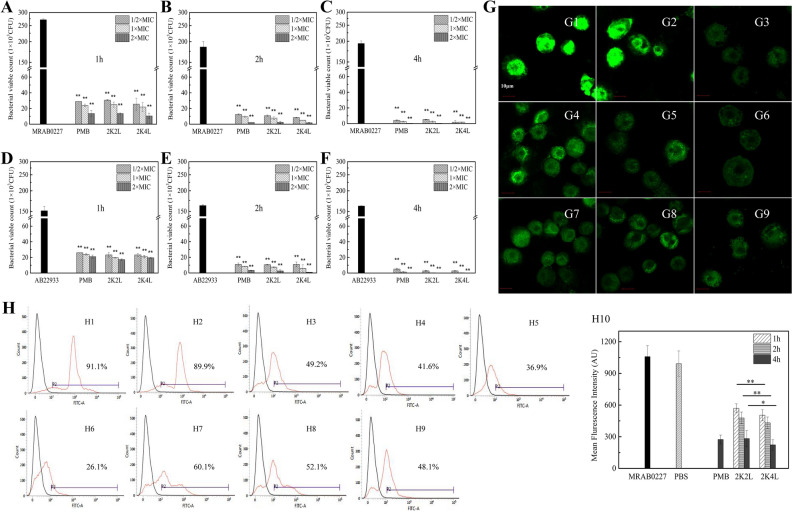
Bactericidal activity of the peptides against intracellular *A. baumannii* in THP-1 cells. (**A–C**) Bacterial viable count of MRAB 0227 in THP-1 cells treated with the peptides for 1 h (**A**), 2 h (**B**) and 4 h (**C**). (**D–F**) Bacterial viable count of AB 22933 in THP-1 cells treated with the peptides for 1 h (**D**), 2 h (**E**) and 4 h (**F**). (**G**) Laser scanning confocal microscopy images of MRAB 0227 in THP-1 cells (**G1**: MRAB 0227; **G2**: PBS; **G3**: PMB-4 h; **G4**: 2K4L-1 h; **G5**: 2K4L-2 h; **G6**: 2K4L-4 h; **G7**: 2K2L-1 h; **G8**: 2K2L-2 h; **G9**: 2K2L-4 h). *(P < 0.05) and **(P < 0.01) indicate statistically significant differences between peptide and the MRAB 0227/AB 22933 group. (**H**) Analysis of MRAB 0227 uptake into THP-1 cells by flow cytometry (**H1**: MRAB 0227; **H2**: PBS; **H3**: PMB-4 h; **H4**: 2K4L-1 h; **H5**: 2K4L-2 h; **H6**: 2K4L-4 h; **H7**: 2K2L-1 h; **H8**: 2K2L-2 h; **H9**: 2K2L-4 h) and the mean fluorescence intensity of THP-1 cells (H10). *(P < 0.05) and **(P < 0.01) indicate statistically significant differences between the 2K4L and 2K2L groups.

To evaluate the antibacterial activity of 2K4L against intracellular *A. baumannii*, bacterial numbers were detected in THP-1 cells infected with *A. baumannii* by using the spiral-plating method. After THP-1 cells (1 × 10^6^ cells/well) were incubated with mid-log phase* A. baumannii* (1 × 10^7^ CFU/mL) for 2 h, the numbers of MRAB 0227 and AB 22933 within the cells were 3.9 × 10^5^ and 2.9 × 10^5^ CFU/cell, respectively. Treatment with the peptides for 1–4 h, consistent with the positive control PMB, significantly reduced the intracellular bacterial number in a concentration- and time-dependent manner (Fig. 1). The viable numbers of MRAB 0227 (Fig. 1C) and AB 22933 (Fig. 1F) within the THP-1 cells were 205 and 105 CFU/cell after treatment with 2K4L at a concentration of 2 × MIC for 4 h, respectively, which were reduced by more than 99% compared to the control group. 2K2L also reduced the viable number of MRAB 0227 and AB 22933 cells by more than 99% compared to the control group. The results suggested that 2K4L and 2K2L had antibacterial activity against intracellular bacteria. This suggestion was confirmed by confocal laser scanning microscopy images (Fig. 1G) and flow cytometry data (Fig. 1H). As shown in Fig. 1G, the addition of different concentrations of 2K4L and 2K2L and 2 × MIC PMB reduced the number of MRAB 0227 labeled with green fluorescence in THP-1 cells, and the green fluorescence decreased as the peptide concentration increased. Figure 1H3 showed that the percentage of green fluorescence decreased by 49.2% after treating MRAB 0227 with the positive control PMB for 4 h. The percentage of green fluorescence from MRAB 0227 decreased by 41.6, 36.9 and 26.1% upon treatment with 2K4L for 1 h, 2 h and 4 h, respectively (Fig. 1H4–H6), and 60.1, 52.1 and 48.1% upon treatment with 2K2L for 1 h, 2 h and 4 h, respectively (Fig. 1H7–H9). Compared to the control group, the average fluorescence intensity decreased by 95.3% and 92.7% after treatment with 2K4L and 2K2L for 4 h (Fig. 1H10).

**Figure 2:**
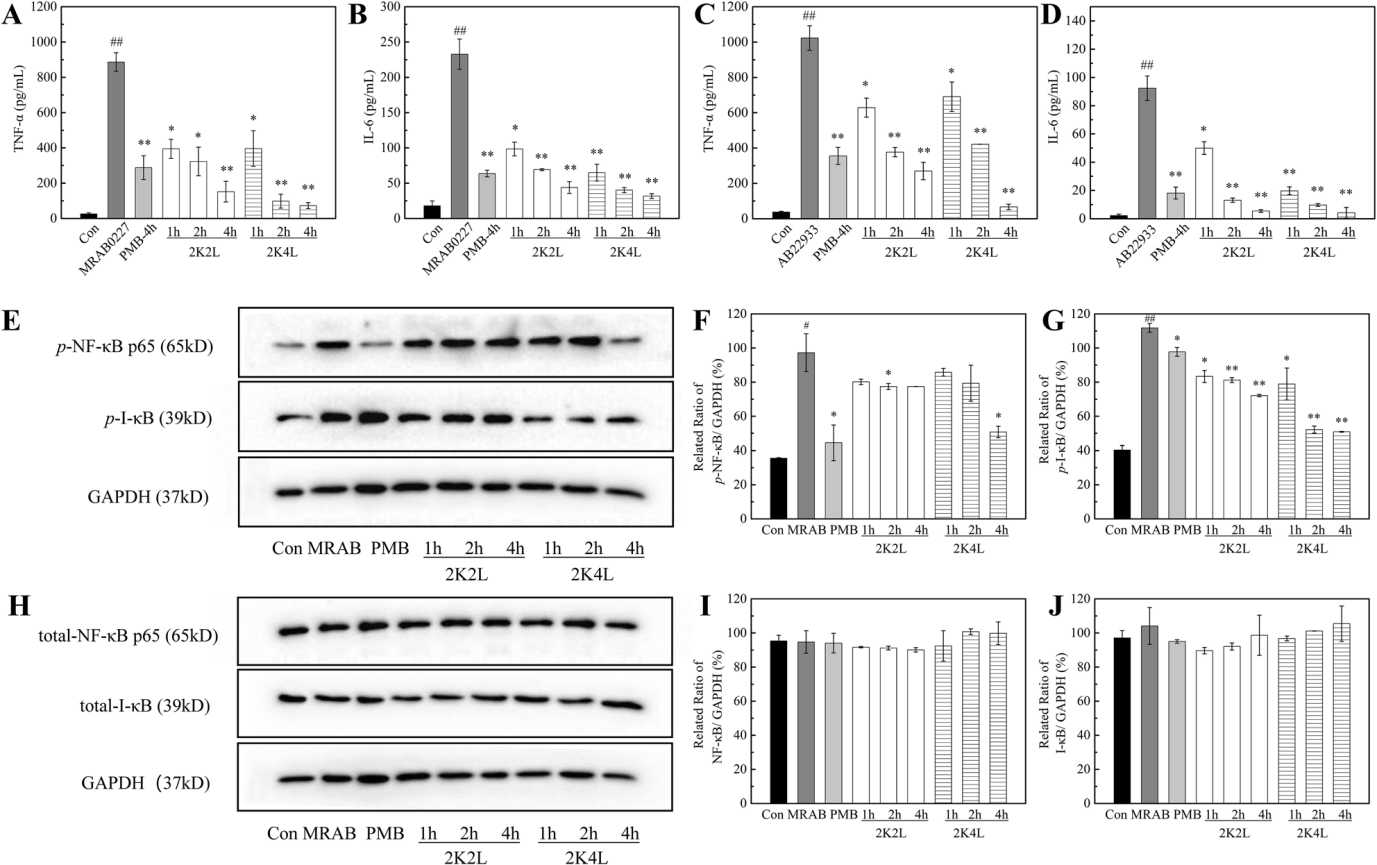
2K4L inhibits the inflammatory response in infected THP-1 cells. (**A–D**) Effect of 2K4L (1, 2 and 4 h) on the production of TNF-α (**A,C**) and IL-6 (**B,D**) by ELISA in THP-1 cells infected with MRAB 0227 (**A,B**) and AB 22933 (**C,D**) for 4 h. (**E–J**) Western blotting analysis of the effects of 2K4L on the expression of *p*-NF-κB (**E,F**), *p*-IκB (**E,G**), total-NF-κB (**H,I**) and total-IκB (**H,J**) after the cells were infected with MRAB 0227 for 4 h and incubated with or without peptides for 1, 2 and 4 h. ^#^(P < 0.05) and ^##^(P < 0.01) indicate statistically significant differences between the MRAB 0227/AB 22933 group and control (0.9% NaCl). PMB-treated MRAB 0227/AB 22933 group for 4 h as a positive control. *(P < 0.05) and **(P < 0.01) indicate statistically significant differences between the peptide and MRAB 0227/AB 22933 groups.

*A. baumannii* induced the inflammatory response in THP-1 cells. As shown in Fig. 2A–D, infection of THP-1 cells with *A.baumannii* significantly induced cellular production of TNF-α and IL-6, and the positive control PMB significantly inhibited the production of inflammatory factors. As shown in Fig. 2A,B, the releases of TNF-α and IL-6 significantly increased in the THP-1 cells infected with MRAB 0227 for 4 h, but 2K4L and 2K2L reduced the production of TNF-α and IL-6 in a time-dependent manner, decreased by 91.7% and 82.9% for TNF-α, and by 86.4% and 81.7% for IL-6 in THP-1 cells treated with the peptides for 4 h, respectively. AB 22933 also induced the inflammatory response in THP-1 cells, 2K4L and 2K2L reduced the releases of TNF-α by 93.5% and 73.6%, and by 95.5% and 89.2% for IL-6 in THP-1 cells treated with the peptides for 4 h, respectively (Fig. 2C,D). The NF-κB signaling pathway is a classic pathway that regulates inflammation. Compared with the control group, MRAB 0227 significantly increased the expression levels of phosphorylated NF-κB p65 (*p*-NF-κB) and IκB (*p*-IκB) (Fig. 2E–G) but had no effect on the expression of nonphosphorylated NF-κB p65 and IκB (Fig. 2H–J). The expression levels of *p*-IκB and *p*-NF-κB were downregulated by 50% after 4 h of 2K4L administration, suggesting that 2K4L inhibited the inflammatory response by downregulating the phosphorylation levels of the signaling proteins NF-κB p65 and IκB in MRAB 0227-infected THP-1 cells.

### Bactericidal activity of 2K4L against *A. baumannii* in *C. elegans*

**Figure 3:**
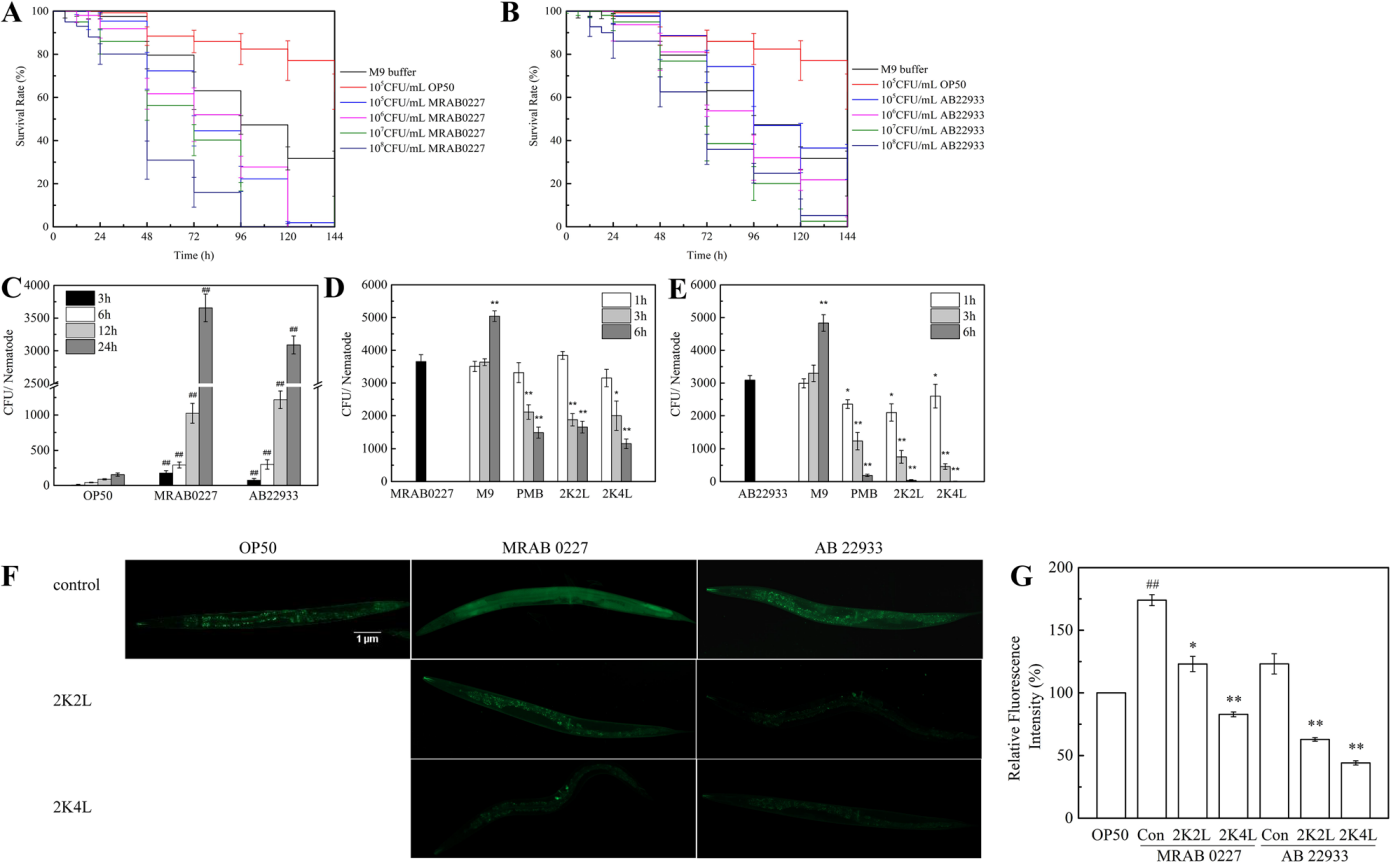
Bactericidal activity of 2K4L against *A. baumannii* in *C. elegans.* (**A,B**) *C. elegans* was placed on NGM with either *E. coli* OP50 or *A. baumannii* (**A**: MRAB 0227; **B**: AB 22933) for 0–144 h, and the survival rate was monitored every 6 h on the first day and at 24 h intervals after a day. (**C–E**) Live bacteria colonization of *C. elegans*. (**C**) The live bacteria colonization in *C. elegans* with *A. baumannii* infection for 3, 6, 12 and 24 h. (**D,E**) Antibacterial effect of 2K4L for 1, 3 and 6 h on *C. elegans* exposed to MRAB 0227 (**D**) and AB 22933 (**E**) for 24 h. (**F,G**) Fluorescence microscopy images of *C. elegans* fed with FITC-D-Lys labeled-*A. baumannii* versus OP50 (**F**). Quantification of FITC-D-Lys (green fluorescence) in worms (**G**). ^##^(P < 0.01) indicates statistically significant differences between the OP50 group and MRAB 0227/AB 22933 group. *(P < 0.05) and **(P < 0.01) indicate statistically significant differences between the peptide and MRAB 0227/AB 22933 groups.

First, the effect of *A. baumannii* on nematode survival was investigated. The killing experiment was designed to mimic the conditions under which a standard nematode lifespan would be measured with the animals feeding on the laboratory standard food source of *E. coli* OP50 alone. The results showed that compared to the control (*E. coli* OP50), the survival rate of nematodes significantly decreased in a time- and concentration-dependent manner after they were exposed to multidrug-resistant *A. baumannii* strain MRAB 0227 (Fig. 3A) and sensitive *A. baumannii* strain AB 0227 (Fig. 3B). All worms were dead at 96 h and 120 h of exposure to 1 × 10^8^ CFU/mL MRAB 0227 and AB 22933, respectively. Nematode death caused by bacterial infection can be divided into slow killing and fast killing^14^. As shown in Fig. 3A,B, *A. baumannii* killed *C. elegans* in a slow killing manner because the survival rate of nematodes was more than 80% after exposure to *A. baumannii* for 0–24 h. The nematodes exposed to 1 × 10^8^ CFU/mL *A. baumannii* for 24 h were used in the following experiments.

Pathogenic bacteria can colonize the intestine of worms, causing disease and even death of nematodes^15^. Here, the colonization of *A. baumannii* was first investigated by calculating the viable colony number of bacteria at a given time point (3, 6, 12 and 24 h after initial exposure). As shown in Fig. 3C, the numbers of colonies in the intestine of worms exposed to MRAB 0227 and AB 22933 increased from 154 to 3655 CFU and 3090 CFU, respectively. The numbers of colonies in the intestine of worms significantly decreased in a time-dependent manner after PMB and the peptides treatment. After 2K4L treatment for 6 h of the nematodes exposed to MRAB 0227 (Fig. 3D), the number of viable colonies was reduced by 77.3% to 1146 CFU per worm, while that in the 2K2L group was reduced by 67.2% to 1653 CFU per worm. After treatment with 2K4L and 2K2L for 6 h, the number of viable colonies in nematodes infected with AB 22933 was almost 0 CFU (Fig. 3E), suggesting that the peptides 2K4L and 2K2L showed stronger antibacterial ability against AB 22933 than against MRAB 0227.

The antibacterial activity of the peptides on *A. baumannii* (labeled with FITC-D-Lys) in nematodes was observed by live cell fluorescence microscope. As shown in Fig. 3F, the green fluorescence from the lysed *E. coli* OP50 was mainly concentrated in the worm intestinal tract; however, the green fluorescence was distributed in the whole body of the worms exposed to MRAB 0227 or AB 22933, and the fluorescence intensity of the worms exposed to MRAB 0227 and AB 22933 was 174% and 123% that of the worms fed OP50 alone (Fig. 3G). The peptide 2K4L decreased the fluorescence intensity of the worms exposed to MRAB 0227 and AB 22933 by 52% and 64%, and 2K2L decreased the fluorescence intensity by 29% and 49%, respectively.

### Protective action of 2K4L on *C. elegans*

**Figure 4:**
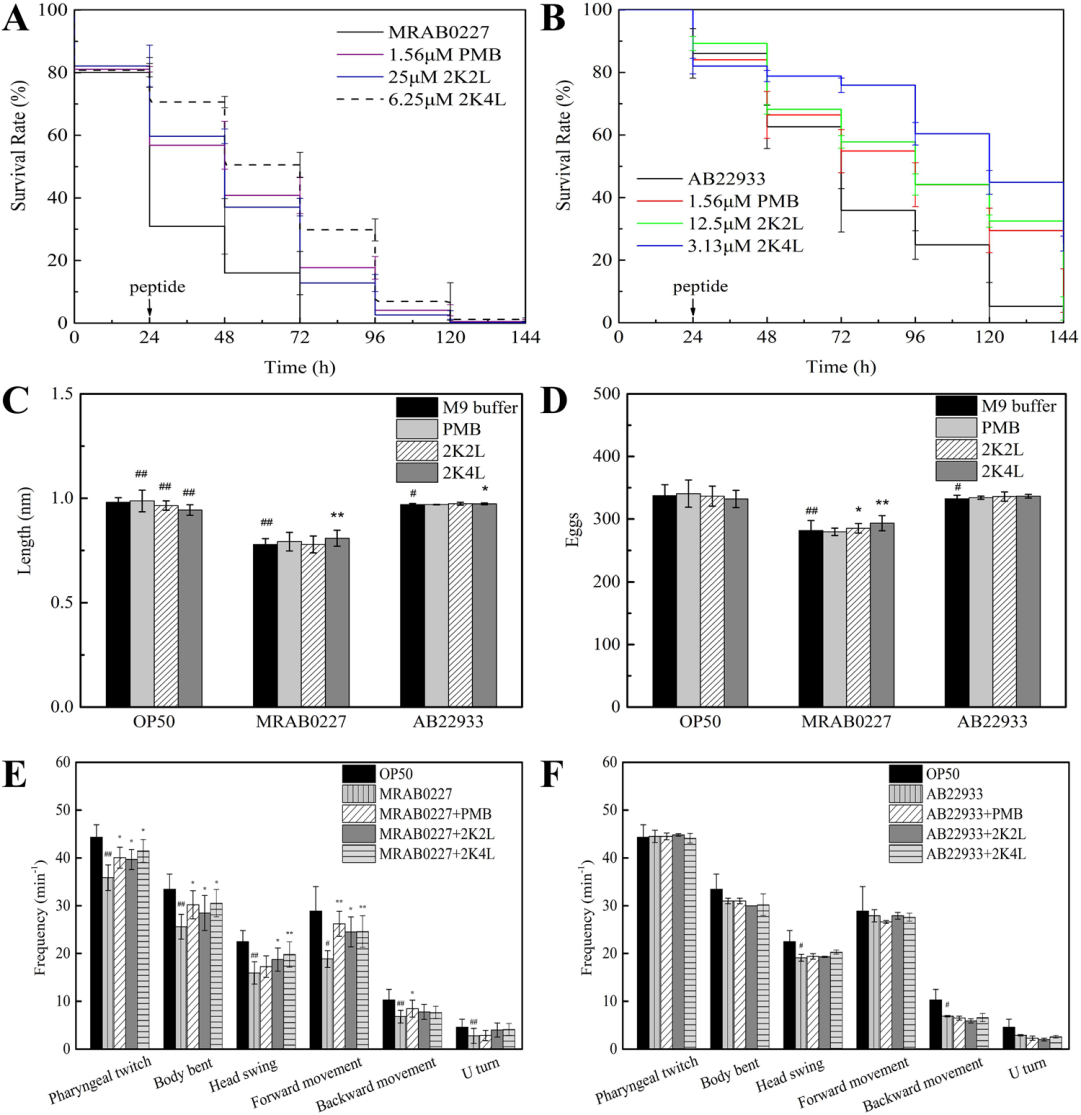
2K4L at a concentration of 6.25 μM extended the lifespan and healthspan of *C. elegans* exposed to *A. baumannii* for 24 h. (**A,B**) The effect of 2K4L on the survival rate of *C. elegans* induced by *A. baumannii* (**A**: MRAB 0227; **B**: AB 22933). (**C,D**) The effect of 2K4L on growth ability (**C**) and reproductive ability (**D**). (**E,F**) Behavioral phenotypes (pharyngeal twitch, body bent, head swing, forward movement, backword movement and U turn) of *C. elegans* (**E**: MRAB 0227; **F**: AB 22933). ^#^(P < 0.05) and ^##^(P < 0.01) indicate statistically significant differences between the OP50 group and MRAB 0227/AB 22933 group. *(P < 0.05) and **(P < 0.01) indicate statistically significant differences between the peptide and MRAB 0227/AB 22933 groups.

The protective effect of the peptides on *C. elegans*, including lifespan, growth, reproduction, and behavioral phenotypes, was investigated. As shown in Fig. 4A, treatment with 2K4L and 2K2L for 24 h increased the survival rate of nematodes from 30.9 to 70.6% and 59.71% after L4-stage worms were exposed to MRAB 0227 for 24 h. All nematodes exposed to MRAB 0227 in the absence or presence of the peptides survived 0 and 13–30% at 96 h, respectively. All nematodes exposed to AB 22933 died at 144 h in the absence of peptide, while the nematodes survived 47.9% and 32.5% after treatment with 2K4L and 2K2L, respectively (Fig. 4B).

The growth, reproduction and behavioral phenotypes of the nematodes were determined to evaluate their physiological indices. Figure 4C shows that the body length of worms exposed to MRAB 0227 and AB 22933 was reduced by 20.6% and 1% compared to the control group (OP50), respectively. PMB could promote the nematode growth. 2K4L increased the body length of nematodes exposed to MRAB 0227 and AB 22933 by 3.8% and 0.3%, respectively. 2K2L showed no significant effect on the body length of nematodes exposed to *A. baumannii*. As shown in Fig. 4D, MRAB 0227 and AB 22933 decreased the oviposition of nematodes. Each worm produced 11–12 eggs after the addition of 2K4L and 3–4 eggs after the addition of 2K2L compared to the group exposed to MRAB 0227. While the nematode oviposition ability had no effect by positive control PMB.

The behavioral phenotypes of the nematodes were recorded, including the frequency of pharyngeal twitch, body bent, head swing, forward movement, backward movement and U-turn. As shown in Fig. 4E, all movement frequencies of the nematodes treated with MRAB 0227 were significantly delayed compared to the control group (OP50); however, the addition of 2K4L and 2K2L markedly increased the frequencies of pharyngeal twitch, body bent, head swing and forward movement and had no effect on backward movement and U-turn of the nematodes. The peptides did not significantly improve all movement of the nematodes exposed to AB 22933 (Fig. 4F). The result of the positive control PMB was consistent with that of AMPs.

In conclusion, 2K4L could improve the lifespan of nematodes exposed to pathogenic bacteria and exhibit protection by improving growth, reproduction and behavioral phenotypes.

### 2K4L reduces the oxidative stress response in *C. elegans* by inhibiting the p38 MAPK-mediated immune pathway

**Figure 5:**
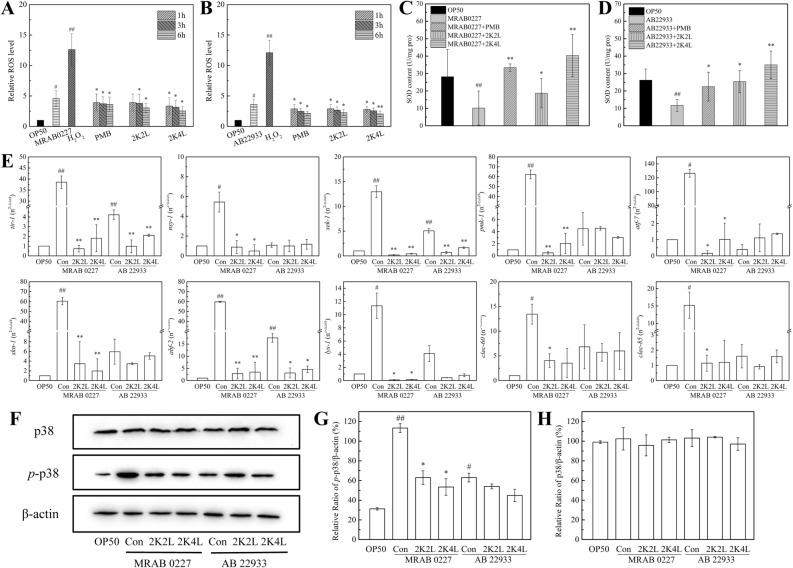
2K4L ameliorated oxidative stress in *C. elegans* induced by *A. baumannii* and promoted innate immunity of *C. elegans* through the p38 MAPK/PMK-1 pathway. (**A,B**) Quantitation of intracellular levels of ROS production in *C. elegans* by DCFH-DA assay (**A**: MRAB 0227; **B**: AB 22933). The results are expressed as the percentage of fluorescence (% DCF) relative to the OP50 group, which is set as 1. (**C,D**) The level of superoxide dismutase (SOD) was measured in *C. elegans* (**C**: MRAB 0227; **D**: AB 22933). (**E**) Expression of immune response genes in *C. elegans* was detected by RT‒qPCR. (**F–H**) Western blotting analysis of the effects of 2K4L on the protein expression of *p*-p38 and p38. ^#^(P < 0.05) and ^##^(P < 0.01) indicate statistically significant differences between the OP50 group and MRAB 0227/AB 22933 group. *(P < 0.05) and **(P < 0.01) indicate statistically significant differences between the peptide and MRAB 0227/AB 22933 groups.

Bacterial pathogens cause an oxidative stress response, resulting in ROS production^16^. As shown in Fig. 5A, the ROS levels were significantly increased in *C. elegans* exposed to MRAB 0227 and AB 22933 compared to the control group (OP50 group), increasing by 4.6-fold and 3.6-fold, respectively, but significantly lower than the positive control group (H_2_O_2_ group). The positive control PMB and the peptides significantly reduced ROS reduction in a time-dependent manner. Compared to the MRAB 0227 group (OP50 group), treatment with 2K4L and 2K2L for 1 h decreased the ROS levels by 27% and 14.6%, respectively, and decreased them by 44.7% and 33.5% at 6 h, respectively. The peptides showed similar effects on the decrease in the ROS level of *C. elegans* exposed to AB 22933 (Fig. 5B).

SOD is another important index to evaluate the oxidative stress response in the first line of defense against oxidative damage^17^. As shown in Fig. 5C, SOD content was approximately 28 U/mg protein in the control group (OP50 group) but significantly decreased to 10 U/mg protein after infection with MRAB 0227. Treatment with 2K4L and 2K2L for 6 h increased the SOD content to 40.3 and 18.7 U/mg protein, respectively. The peptides also improved the SOD levels of the AB 22933 group (Fig. 5D). The positive control PMB treatment significantly increased the SOD content to 33.4 and 22.5 U/mg protein of the MRAB 0227 group and AB 22933 group, respectively. These results indicated that 2K4L could reduce the oxidative stress response induced by MRAB 0227 and AB 22933 infection in nematodes.

The oxidative stress response can activate the p38 MAPK/PMK-1 signaling pathway in nematodes^18^. Here, the gene expression of the p38 MAPK/PMK-1 signaling proteins (*nsy-1*, *tir-1*, *sek-1* and *pmk-1*), previously identified AMPs (*abf-2*, *clec-60* and *clec-85*), and other transcription factors (*skn-1* and *atf-7*) was measured by RT‒qPCR. As shown in Fig. 5E, MRAB 0227 significantly increased the expression of genes in the p38 MAPK signaling pathway, resulting in the activation of the innate immune response of *C. elegans*. The gene expression of the transcriptional activator *atf-7* was upregulated 126-fold compared to the control group (OP50) and upregulated 60-fold for *pmk-1*, *skn-1* and *abf-2*. The peptides 2K4L and 2K2L significantly inhibited the expression of these genes, with inhibition above 95%. AB 22933 increased the expression of the *tir-1*, *sek-1* and *abf-2* genes (4–17-fold), and the peptides significantly inhibited the expression of these genes. Western blot results showed that MRAB 0227 and AB 22933 significantly elevated the levels of phosphorylated p38; however, 2K4L remarkably suppressed the expression of phosphorylated p38 by 47% and 40% in nematodes infected with MRAB 0227 and AB 22933, respectively (Fig. 5F,G). The expression of total p38 showed no change in *A. baumannii-*infected nematodes and peptide-treated nematodes (Fig. 5H). The results suggested that 2K4L and 2K2L showed no effect on the total p38 protein levels in *A. baumannii*-exposed *C. elegans*, but increased the phosphorylation of p38, resulting in the activation of p38/MAPK signaling pathway and the increase of the expression of downstream genes.

### Protective action of 2K4L on septic mice induced by LPS

**Figure 6:**
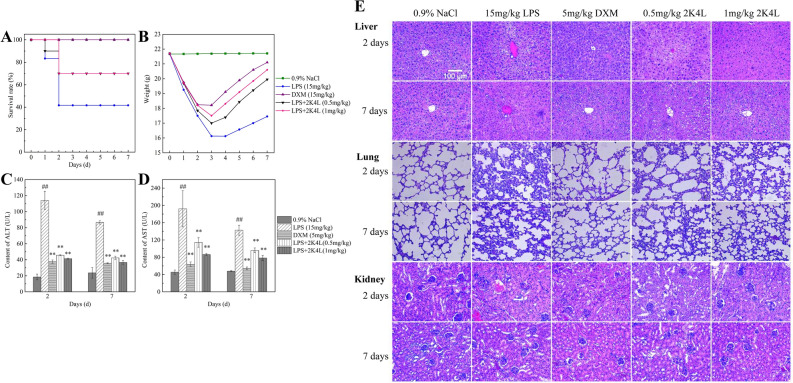
2K4L had a protective effect on septic mice infected with LPS. (**A**) Survival rate of the septic mice induced by LPS. (**B**) Body weight of the septic mice induced by LPS. (**C,D**) Liver function of the septic mice induced by LPS. (**E**) Pathological observation of the liver, lung and kidney of the septic mice induced by LPS (original magnification × 200, n = 3). ^#^(P < 0.05) and ^##^(P < 0.01) indicate statistically significant differences between the LPS group and the control (0.9% NaCl). *(P < 0.05) and **(P < 0.01) indicate statistically significant differences between the peptide and LPS groups.

LPS, named endotoxin, is a critical factor that induces severe and systemic inflammatory responses and acute tissue injury in organs such as the lung, liver and kidney during sepsis. In the present study, we further investigated the anti-inflammatory effects of 2K4L on LPS-induced septic mice. As shown in Fig. 6A, the survival rate of septic mice was 40% of that of the blank group after 2 days of LPS injection. Doses of 0.5 mg/kg and 1 mg/kg 2K4L increased the survival rate of septic mice by 70%. DMX, a positive control, increased survival by 100%. The body weight change curves are shown in Fig. 6B within 7 days after LPS injection. Compared with the LPS injection group, 2K4L improved the weight loss of mice in a dose-dependent manner. DMX exhibited the same effect. Figure 6C,D show the levels of ALT and AST. The levels of ALT and AST in LPS-induced septic mice were 6.2-fold and 4.2-fold higher than those in the blank control group (0.9% NaCl). 2K4L significantly inhibited ALT and AST levels in a concentration-dependent manner. Compared with the LPS group, 2K4L at 1 mg/kg inhibited ALT by 63.7% and 67.8% on Days 2 and 7 and inhibited AST by 55.1% and 59.3% on Days 2 and 7, respectively. DMX inhibited ALT by 67% and 58.8% on Days 2 and 7 and inhibited AST by 66.9% and 62% on Days 2 and 7, respectively. The livers, lungs and kidneys of mice treated with 2K4L for 2 days and 7 days were collected for histochemical analysis. As shown in Fig. 6E, severe tissue damage was observed in the LPS-treated group. The liver tissues showed swelling, necrosis and irregular distribution. Obvious congestion and inflammatory cell infiltration of the liver sinusoid were observed. Lung tissue injury was characterized by alveolar defects and significant thickening of the alveolar septum. Inflammatory cells were increased, and tissues were deformed. Kidney injury manifests as a glomerulus that increases in volume and changes in shape. The glomerular capillaries were dilated and filled with blood cells. The glomerular sacs shrank in space and even adhered to the glomeruli. The damage to liver, lung and kidney tissue was improved after 2 days of 2K4L administration. In the liver, the distribution of hepatocytes was significantly uniform, with reduced congestion and inflammatory cells. The number of inflammatory cells in lung and kidney tissue increased, and the tissue morphology recovered. The tissues were significantly reduced after 7 days of administration compared with 2 days. These results indicated that 2K4L could protect septic mouse organs from damage caused by LPS.

### 2K4L alleviates the inflammatory response by the NF-κB signaling pathway in LPS-induced septic mice

**Figure 7:**
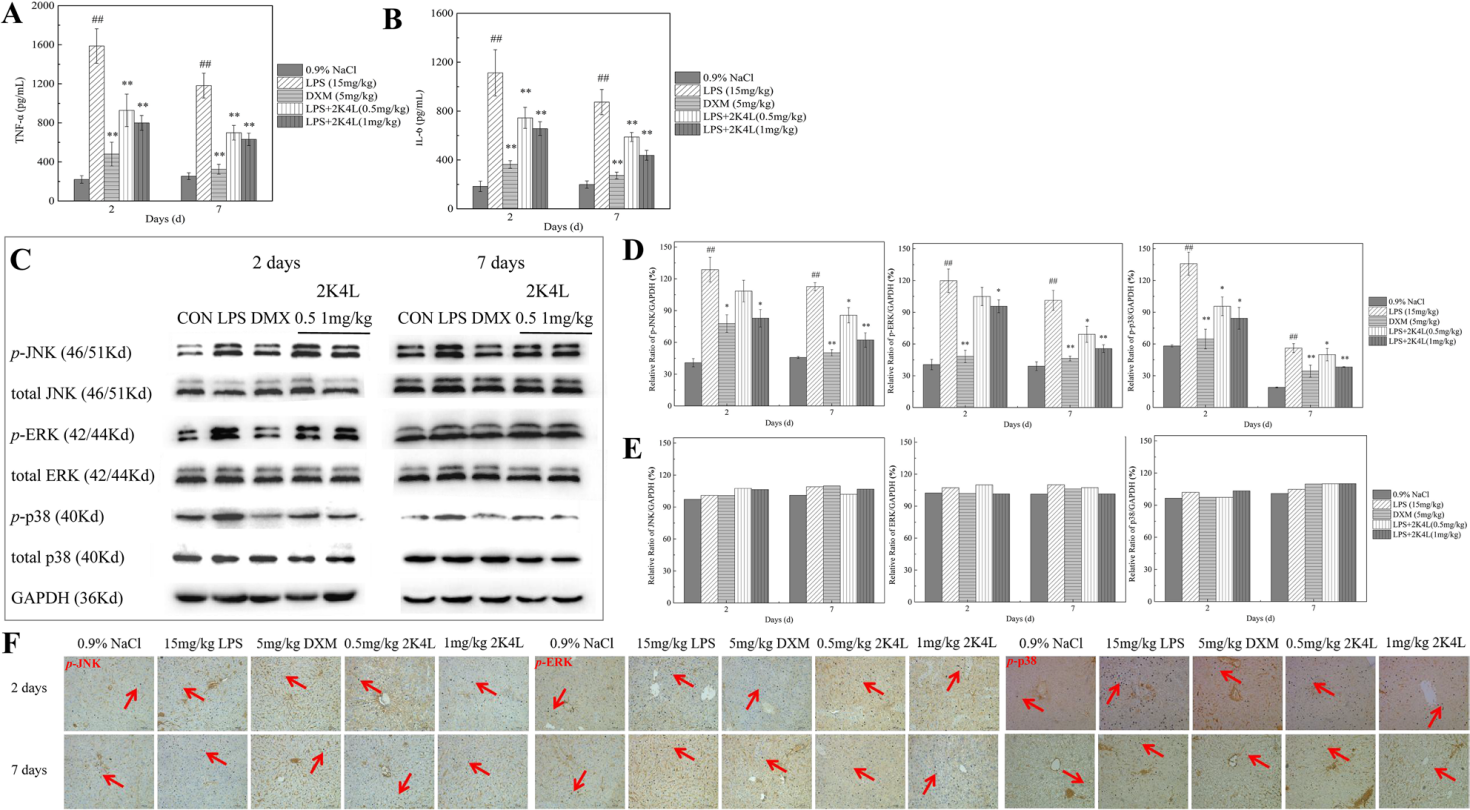
2K4L could inhibit inflammation in septic mice infected by LPS through the MAPK pathway. (**A**) Effect of 2K4L on the production of TNF-α in the serum of septic mice induced by LPS. (**B**) Effect of 2K4L on the production of IL-6 in the serum of septic mice induced by LPS. (**C–E**) Effect of 2K4L on the MAPK inflammatory pathway of total JNK, *p*-JNK, total ERK, *p*-ERK, total p38 and *p*-p38 in septic mice induced by LPS. (**F**) Immunohistochemical analysis of phosphorylated JNK, ERK and p38 protein in the livers of septic mice induced by LPS. (**H–I**) Effect of 2K4L on the NF-κB inflammatory pathway of p-IκB and p-NF-κB in septic mice induced by LPS (original magnification × 200, n = 3). ^#^(P < 0.05) and ^##^(P < 0.01) indicate statistically significant differences between the LPS group and the control (0.9% NaCl). *(P < 0.05) and **(P < 0.01) indicate statistically significant differences between the peptide and LPS groups.

As shown in Fig. 7A,B, serum TNF-α and IL-6 were significantly increased in MRAB 0227-induced septic mice. 2K4L significantly decreased the release of TNF-α and IL-6 in a time- and dose-dependent manner. On Days 2 and 7 of 2K4L administration at 0.5 mg/kg, the expression of TNF-α was downregulated by 41.5% and 55.9%, and the expression of IL-6 was downregulated by 33.1% and 47.2%, respectively. 2K4L at a dose of 1 mg/kg downregulated the expression of TNF-α by 49.7% and 60.2% and by 67.4% and 75.5% for IL-6, respectively.

To further examine the effect of 2K4L on the inflammatory signaling pathway of MAPKs, the expression of p38, ERK and JNK proteins in the liver tissue of LPS-induced septic mice was detected by western blotting. As shown in Fig. 7C, the expression levels of phosphorylated p38 (*p*-p38), ERK (*p*-ERK) and JNK (*p*-JNK) in the liver tissue of LPS-induced mice were significantly increased compared with those in the control group (0.9% NaCl). On Days 2 and 7 of 2K4L administration at 0.5 mg/kg, the expression of *p*-p38 was downregulated by 29.6% and 63.3%, *p*-ERK was downregulated by 12.5% and 42.3%, and *p*-JNK was downregulated by 15.7% and 33.5%, respectively. As the dose of 2K4L administration increased to 1 mg/kg body weight, the expression of *p*-p38 was downregulated by 38% and 71.8%; *p*-ERK was downregulated by 20.1% and 53.6%; and *p*-JNK was downregulated by 35.6% and 51.6%, respectively. However, the expression levels of nonphosphorylated p38, ERK and JNK were not significantly different from those in the blank group and LPS group (Fig. 7D,E). This result indicated that 2K4L only inhibited the phosphorylation levels of MAPK signaling proteins in LPS-induced septic mice (Supplementary Information). The immunohistochemistry data showed similar results. As shown in Fig. 7F, LPS significantly induced the expression of *p*-p38, *p*-ERK and *p*-JNK (red arrow) in the liver tissues of septic mice, and 2K4L reduced the expression levels of three phosphorylated proteins after 2 days of 2K4L administration. The dose of 1 mg/kg 2K4L exhibited better inhibition than that of 0.5 mg/kg 2K4L.

**Figure 8:**
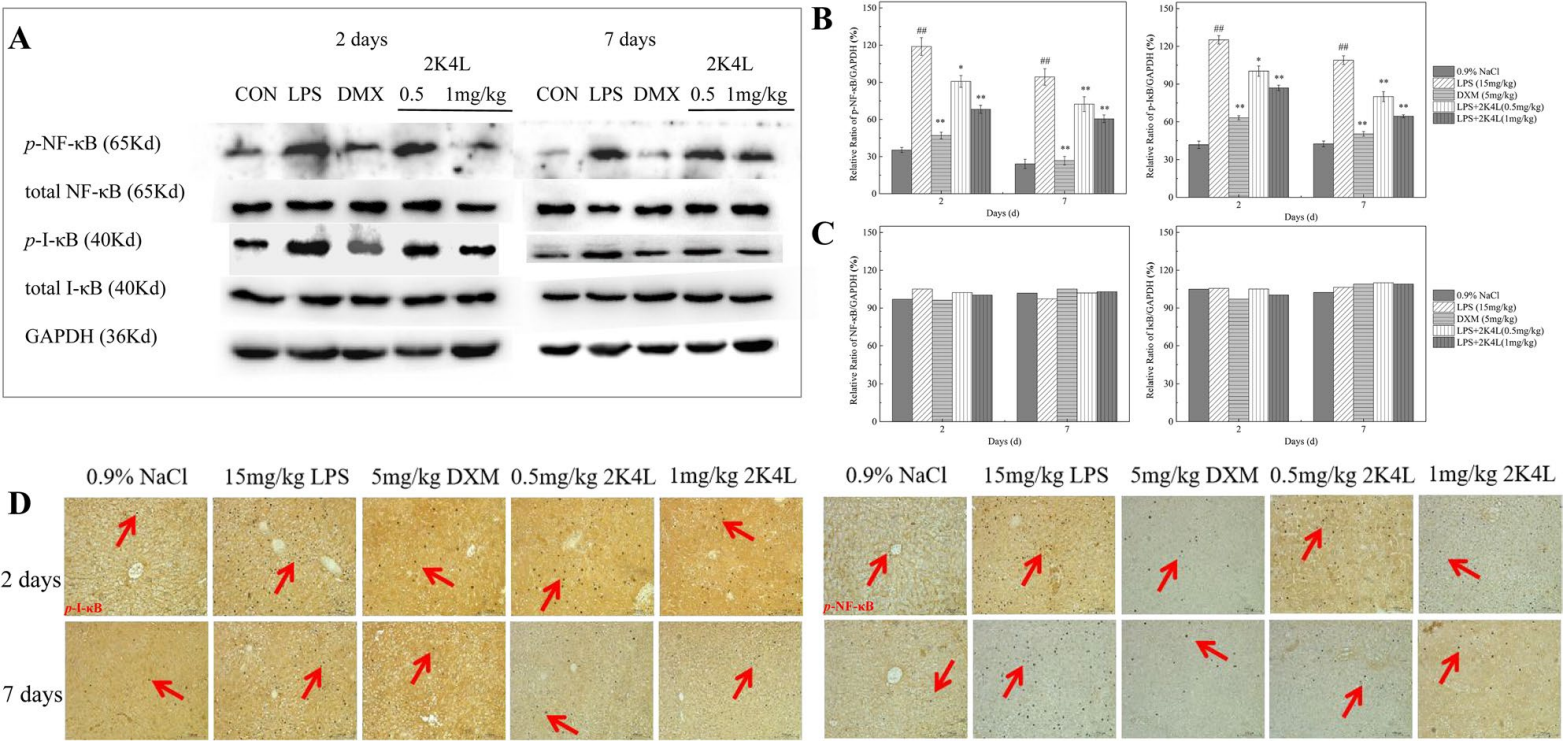
2K4L could inhibit inflammation in septic mice infected by LPS through the NF-κB pathway. (**A–C**) Effect of 2K4L on the MAPK inflammatory pathway of total IκB, NF-κB and phosphorylated IκB, NF-κB in septic mice induced by LPS. (**D**) Immunohistochemical analysis of phosphorylated IκB and NF-κB protein in the livers of septic mice induced by LPS (original magnification × 200, n = 3). ^#^(P < 0.05) and ^##^(P < 0.01) indicate statistically significant differences between the LPS group and the control (0.9% NaCl). *(P < 0.05) and **(P < 0.01) indicate statistically significant differences between the peptide and LPS groups.

The effect of 2K4L on the inflammatory signaling pathway of NF-κB was investigated. As shown in Fig. 8A, compared with the control group (0.9% NaCl), the expression levels of phosphorylated IκB (*p*-IκB) and NF-κB p65 (*p*-NF-κB p65) in the liver tissue of LPS-induced septic mice markedly increased by threefold and 3.4-fold over the blank group, respectively. On Days 2 and 7 of 2K4L administration at a dose of 1 mg/kg body weight, the expression of *p*-IκB was downregulated by 19.8% and 36.1%, and *p*-NF-κB p65 decreased by 23.7% and 39.1%, respectively. As the dose of 2K4L administration increased to 1 mg/kg, the expression of *p*-IκB was downregulated by 30.5% and 48.5%, and *p*-NF-κB p65 was downregulated by 42.7% and 49.1%, respectively. Figure 8B,C show that the expression levels of nonphosphorylated IκB and NF-κB p65 were not significantly changed compared to those in the blank group and LPS group, suggesting that 2K4L only inhibited the phosphorylation of NF-κB signaling proteins in LPS-induced septic mice. The mmunohistochemistry results are shown in Fig. 8D. LPS significantly increased the expression of *p*-IκB and *p*-NF-κB, and 2K4L reduced the expression levels of the two phosphorylated proteins after 2 days of 2K4L administration.

## Discussion

It is well known that antimicrobial peptides not only have broad-spectrum antibacterial, antifungal and antiviral activities but also have the ability to favorably modulate immune responses in various organisms^19,20^. 2K4L, as an α-helical structure consisting of four positively charged lysine residues and an amidation of the carboxyl terminal, exhibited broad-spectrum antibacterial activity against gram-negative and gram-positive bacteria. In the current study, we demonstrated that 2K4L could significantly reduce the release of the proinflammatory factors TNF-α and IL-6 by downregulating the phosphorylation levels of NF-κB and MAPK signaling proteins in *A. baumannii*-infected macrophages. We identified that 2K4L could inhibit the inflammatory response induced by *A. baumannii* in *C. elegans* by down-regulating the phosphorylation levels of the PMK-1 gene as well as p38 signaling proteins in the p38 MAPK/PMK-1 signaling pathway. In vivo, 2K4L had protected against LPS-induced shock in mice by modulating inflammatory and immune responses.

LPS is an important main component of the outer membrane of Gram-negative bacteria and is one of the main triggers of the inflammatory response for sepsis and endotoxic shock. Previous investigations have shown that the neutralization of AMPs with LPS might be one of the anti-inflammatory actions of AMPs^21,22^. D-Mt6, designed from the hemlymphocytes of housefly larvae, showed great immunoregulatory activity against LPS-induced inflammation through its LPS neutralization and suppression of MAPK signaling^23^. In addition to the neutralization of LPS, AMPs could intervene in Toll-like receptor (TLR) signaling pathways with potent antiendotoxin properties^24^. The study has found that LL-37 acts as an anti-inflammatory activator and prevents inflammatory activation via TLR2 and TLR4^24^. Our previous data confirmed that 2K4L interacted with LPS^25^. 2K4L could bind with the LPS head group through electrostatic interactions, which caused 2K4L to insert into the acyl chain of lipopolysaccharides through hydrophobic interactions, leading to a significant perturbation of the LPS packing organization. Meanwhile, FITC-labeled 2K4L fluorescence and DLS measurements clearly showed that 2K4L could dissociate the aggregated state of LPS. These results suggested that the anti-inflammatory mechanism of 2K4L may be associated with its interaction with LPS.

AMPs have a series of problems in vivo, such as poor stability, strong toxicity and other side effects, which seriously limit and hinder their conversion and application. Although the anti-inflammatory activity and mode of action of AMPs in vitro have been demonstrated^26^, the anti-inflammatory activity is not yet well understood in vivo. Here, AMP 2K4L showed high anti-inflammatory activity in *Caenorhabditis elegans*.* C. elegans,* as a model organism, has been widely used in the study of pathogenic microorganisms and host immunity and in the development of new drugs^27^. *A. baumannii* is one of the most multidrug-resistant pathogens of the ESKAPE group and has been experimentally proven to be pathogenic and lethal to *C. elegans*^27,28^. Our data also showed that either the multidrug-resistant or sensitive *A. baumannii* (MRAB 0227 or AB 22933) strain inhibited nematode growth and oviposition and delayed mobility, resulting in a shortened lifespan of *C. elegans*. However, 2K4L exerted significant protection to improve the growth, reproduction and behavioral phenotypes of nematodes by killing MRAB 0227 or AB 22933 in the intestinal lumen of *C. elegans*. Pathogenic microbes can induce ROS, acting as signaling molecules to activate cytoprotective pathways, resulting in host tissue damage early in infection and later leading to organismal failure and death in *C. elegans*^29^. In addition to antibacterial activity, some AMPs also have potent antioxidant effects^16,30^. Fractions of SDSP from dogfish skin showed in vitro antioxidant activities that significantly improved motility, reduced ROS and H_2_O_2_ levels in *C. elegans*, and increased its resistance to oxidative stress^31^. Our data showed that 2K4L could significantly increase SOD activity and inhibit ROS levels induced by *A. baumannii*, avoiding the potential damage caused by oxidative stress.

In organisms, including *C. elegans*, the immune response is activated after infection in response to the pathogen. After exposure to pathogens, *C. elegans* normally elicits an innate immune response against invading pathogens. The centrality of ROS as triggers of host defense responses and as drivers of bacterial pathogenesis in *C. elegans*. ROS sensing leads to activation of the p38 MAPK signaling pathway and the insulin signaling pathway, which results in activation of the transcription factors SKN-1/NRF2, ATF-7/ATF7, and DAF-16/FOXO3. In addition, ROS activate HIF-1/HIF1a, NHR-49/HNF4, and HLH-30/TFEB^16^, enabling host defense against pathogens. Our data showed that MRAB 0227 significantly upregulated the gene expression of antimicrobial peptides (*abf-2*)*,* C-type lectin family genes (*clec-60* and *clec-85*) and lysozyme (*lys-1*) in the p38 MAPK/PMK-1 signaling pathway and downregulated the expression level of *atf-7* (126-fold), a transcriptional regulator that acts as a repressor of effector molecule expression but switches to an activator role upon activation by PMK-1. ATF-7 contributes to protection from pathogenic bacteria^32^. PMK-1 was shown to regulate the innate immune response to pathogen infection and is required to induce somatic stress resistance in response to intestinal infection by *Pseudomonas aeruginosa*^33^. The present study demonstrated that 2K4L could induce the expression level of PMK-1 (60-fold) and downregulate the phosphorylation levels of signaling proteins of p38 in the proinflammatory pathway in *C. elegans*.

MAPK pathways play conserved signaling roles during development and for the transduction of environmental stimuli to generate cellular responses in evolutionarily diverse organisms. For example, the p38 homolog PMK-1 is the terminal MAPK of a stress- and immune-responsive signaling pathway^34^. In addition, the main regulatory mechanisms of insulin signaling in *C. elegans* are similar and highly conserved to those in mammals. We further examined the immunomodulatory effects of 2K4L in LPS-induced septic shock mice. Indeed, direct evidence has shown that 2K4L significantly increased the survival rate of infected mice. 2K4L, with low toxicity in vivo, alleviates liver function by decreasing ALT and AST levels in serum and protects septic mice by inducing severe damage to mouse liver, lung and kidney tissues caused by MRAB 0227 infection. The present study also demonstrated that 2K4L could reduce the release of TNF-α and IL-6 with significant anti-inflammatory activity in vivo by downregulating the phosphorylation levels of MAPK and NF-κB signaling proteins in the proinflammatory pathway.

In summary, 2K4L displayed potent anti-inflammatory activity in MRAB 0227-induced THP-1 cells and *C. elegans* by the proinflammatory pathway. Most importantly, 2K4L is expected to be developed as a new therapeutic agent for LPS-induced septic shock.

## Methods

### Bacteria, macrophage cell and nematode strain

Multidrug-resistant *Acinetobacter baumannii* (MRAB 0227) strain were collected and identified in the Department of Clinical Laboratory, the First Affiliated Hospital of Dalian Medical University. Standard *Acinetobacter baumannii* CICC 22933 strain (AB 22933) was acquired from China Center for Industrial Culture Collection. Bacterial cells were cultured in Luria Bertani (LB) medium at 37 °C overnight. The human monocytic leukemia cell line THP-1 was obtained from the Cell Bank of the Chinese Academy of Sciences Type Culture Collection Committee (Shanghai, China). THP-1 cells were cultured in RPMI 1640 medium containing 10% fetal bovine serum (FBS) and 0.05 mM β-mercaptoethanol. *Caenorhabditis elegans* N2 (wild-type Bristol isolate) was obtained from the Caenorhabditis Genetics Center (CGC, University of Minnesota, Minneapolis, MN, USA). *C. elegans* was cultured by standard methods^8^. Briefly, *C. elegans* was routinely maintained on nematode growth medium (NGM) agar plates at 20 °C and seeded with freshly prepared *E. coli* OP50 (Uracil deficient *Escherichia coli*) as a food source from CGC. For experiments requiring age-synchronized nematodes, L2 stage *C. elegans* on starved plates was rinsed off, extracted by centrifugation, moved to *E. coli* OP50 seeded plates and raised to the L4 stage worms.

### Bacterial colonization in human macrophage

THP-1 cells (1 × 10^6^ cells/well) were plated into 6-well plates and treated with PMA (100 ng/mL) for 48 h for complete adhesion. After washing twice with serum-free medium, the cells were infected with log-phase MRAB 0227 and AB 22933 cells at concentrations of 1 × 10^5^, 1 × 10^6^ and 1 × 10^7^ CFU/mL and incubated at 37 °C for 1 h, 2 h and 4 h. Extracellular bacteria were removed by washing the plate with PBS, and then THP-1 cells were incubated with the peptides at final concentrations of 1/2 × MIC, 1 × MIC and 2 × MIC for 1 h, 2 h and 4 h at 37 °C. Following incubation, the cells were lysed with 0.1% Triton X-100 for 5 min. The released bacterial suspension was gradiently diluted and spread on agar plates. After incubation overnight at 37 °C, bacterial colonies were counted and recorded.

The bactericidal activity of the peptides in THP-1 cells was examined by using confocal laser scanning microscopy (LSM-710, Carl Zeiss Microimaging, Germany). Briefly, mid-log phase MRAB 0227 cells (1 × 10^7^ CFU/mL) were firstly labeled green fluorescent by staining with the SYTO-9 dye for 30 min at 4 °C in the dark, and then mixed with THP-1 cells (1 × 10^6^ cells/well) and incubated at 37 °C for 2 h. After the suspended bacteria were removed by centrifugation, the cells were treated with the peptides for 1 h, 2 h and 4 h at 37 °C in a 5% CO_2_ incubator. Cells were imaged by using confocal laser scanning microscopy. PMB (Polymyxin B, CAS#:1405-20-5) was used as a positive control. Cells treated with medium alone were used as negative controls.

MRAB 0227-infected THP-1 cells were treated with or without peptides and then digested with trypsin. After being washed with PBS, the cell pellets were collected by centrifugation and resuspended in PBS. The cells were filtered into sample tubes, and the fluorescence intensities of the cells were detected by a BD FACSVerse™ cell analyzer (BD, Mississauga, CA).

### Proinflammatory factor assay

The release of the proinflammatory factors TNF-α and IL-6 was measured either in THP-1 cell cultures or murine blood serum using Invitrogen ELISA kits according to the manufacturer’s instructions (Proteintech Group, Chicago, USA). Briefly, THP-1 cells were first incubated with phorbol 12-myristate 13-acetate (PMA, 100 ng/mL) for 48 h to adhere to the wall and differentiate into macrophages. Then, the cells (1 × 10^6^ cells/well) were treated with log-phase MRAB 0227 cells or AB 22933 (1 × 10^7^ CFU/well) for 4 h. After washing two times with PBS to remove the bacteria in solution, the cells were incubated with the peptides 2K2L (25 μM and 12.5 μM) and 2K4L (12.5 μM and 6.25 μM) for 1 h, 2 h and 4 h, respectively. Next, the supernatants were collected by centrifugation at 1000 rpm, and the release of TNF-α and IL-6 was measured with ELISA kits. PMB (1.56 μM) was used as a positive control. For LPS*-*induced septic mice, the levels of TNF-α and IL-6 in mouse serum were measured with ELISA kits.

### Nematode infection assay

Age-synchronized L4 worms (20 worms/plate) were transferred to NGM plates and infected with MRAB 0227 and AB 22933 (1 × 10^5^, 1 × 10^6^, 1 × 10^7^ and 1 × 10^8^ CFU/mL). After overnight infection, all worms were transferred to fresh NGM plates supplied with *E. coli* OP50. Dead and live nematodes were scored every 6 h on the first day and every 24 h thereafter. Every 48 h, live worms were transferred to fresh NGM plates seeded with *E. coli* OP50 to exclude freshly hatched larvae. A nematode was considered dead when it showed no response to the soft touch of the platinum loop. The nematodes that died by adhering to the wall of the glass plate were excluded from the assay. The number of live nematodes was counted continuously for 6 days. *E. coli* OP50 alone was used as a negative control, and the M9 buffer (KH_2_PO_4_, Na_2_HPO_4_, NaCl and MgSO_4_) group without bacteria was used as a control. There were five parallel samples in each experiment. The assay was repeated three times.

### Bacterial colonization in *C. elegans*

The bactericidal activity of peptides against MRAB 0227 and AB 22933 in *C. elegans* N2 was examined as described^9^. Briefly, age-synchronized L4 worms (20 worms/plate) were infected with the log-phase of MRAB 0227 or AB 22933 (1 × 10^8^ CFU/mL) and incubated at 20 °C for 3 h, 6 h, 12 h and 24 h, respectively. After the surface colonized bacteria were removed with M9 buffer, the nematodes were transferred to new NGM plates and treated with peptides for 1 h, 3 h and 6 h, respectively. Then, the worms were washed with M9 buffer and lysed by vortexing in M9 buffer (containing 400 mg quartz sand) for 2–3 min to fragment them, and the suspended bacteria were collected. The suspended bacteria were serially diluted 10 times, spread on LB solid medium and incubated at 37 °C overnight. The numbers of CFU (colony forming units) were recorded.

For fluorescence microscopy, the age-synchronized L4 worms were incubated with the log phase of FITC-D-Lys (Acmec Biochemical Co., Ltd, Shanghai, China)-labeled MRAB 0227 or AB 22933 (1 × 10^8^ CFU/mL) at 20 °C for 24 h, respectively. After the surface-colonized bacteria were removed with M9 buffer, the nematodes were treated with peptides for 6 h and then observed by live cell microscope (DMi8 S Platform, Leica, Germany). The fluorescence intensity was quantified using ImageJ software.

### Lifespan assay

After infection with the log-phase of MRAB 0227 or AB 22933 (1 × 10^8^ CFU/mL) at 20 °C for 24 h, the age-synchronized L4 worms were transferred to NGM plates coated with 2K2L (25 μM) and 2K4L (6.25 μM). The positive control was PMB (1.56 μM). Live nematodes were counted every 24 h and transferred to fresh NGM plates coated with bacterial solution and AMPs. Two replicates were performed. Twenty to thirty worms were tested for the lifespan assay.

### Behavioral assays

Age-synchronized L4 worms were transferred to sterile slides and killed with water vapor at high temperature. The body length of nematodes was accurately measured by body elongation after death, according to Hasegsawa’s improved method^10^. For fertility analysis, 20 young L4 worms were seeded in the middle of each NGM plate and observed at 2, 6, 24 and 48 h after this initial transfer. Nematodes were transferred daily to fresh NGM plates containing bacteria, and the number of offspring was recorded under a microscope every 6 h until the end of the breeding period according to the method of Thomas^11^. Further locomotor behavior analysis of L4 worms, including the frequency of pharyngeal movement, body bending, head swing, forward movement, backward movement and turning, was carried out under a microscope within 1 min. The same procedure was performed for nematodes treated with the bacteria and the peptides on NGM plates.

### Reactive oxygen species (ROS) assay

Age-synchronized L4 worms were infected with log-phase MRAB 0227 or AB 22933 for 24 h. The surface-colonized bacteria were removed with M9 buffer, and the nematodes were transferred to new NGM plates and treated with the peptides for 6 h. After the nematodes were frozen with liquid nitrogen and homogenized, the supernatant was obtained by centrifugation at 12,000 rpm at 4 °C. Fifty microliters of supernatant was mixed with an equal volume of 2,7-dichlorodihydrofluorescein diacetate (DCFH-DA), and the mean fluorescence intensity (MFI) was measured by a microplate reader (Thermo Scientific Co., Beijing, China) at an excitation wavelength of 504 nm and an emission wavelength of 529 nm after 1 h of incubation. The ROS levels were calculated with the following formula^12^: MFI_Experimental group_/MFI_OP50 group_. Nematodes treated with H_2_O_2_ were used as a negative control.

### Superoxide dismutase (SOD) assay

SOD activity was detected following the instructions of the Total Superoxide Dismutase Assay Kit with WST-8 (Beyotime, Shanghai, China). The manipulation process was performed according to the manufacturer’s instructions.

### Quantitative real-time PCR (qPCR) analysis

Synchronized L4 larval stage nematodes were infected with log-phase MRAB 0227 or AB 22933 for 24 h. After the surface-colonized bacteria were removed with M9 buffer, the nematodes were transferred to new NGM plates and treated with the peptides for 6 h. Total RNA was extracted with TRIzol Reagent (Invitrogen)^13^. cDNA was amplified with a HiScript®II Q RT SuperMix for qPCR (+ gDNA wiper) kit (Vazyme, Nanjing, China). Real-time quantitative PCR was performed in an ABI-Prism 7500 Fast sequence detection system (Applied Biosystems) with TB Green® Premix Ex Taq™ II (Tli RNaseH Plus) (TaKaRa, Dalian, China). Gene expression levels were calculated according to the comparative cycle threshold (Ct) method after normalization with the internal control β-actin. The primers used for this study are shown in Table 1.

**Table 1:** Genes and primer sequences.

Gene	Forward	Reverse	Gene ID
*β-actin*	ATCGTCCTCGACTCTGGAGATG	TCACGTCCAGCCAAGT	11461
*tir-1*	TTGGGTGCACAAAGAGCTGA	GGTCGGTGTCGTTCTGTTCA	175502
*nsy-1*	AGCGGCTCGATCAACAAGAA	CCCATTCCACCGATATGCGA	24104671
*sek-1*	CACTGTTTGGCGACGATGAG	ATTCCGTCCACGTTGCTGAT	181043
*pmk-1*	CGACTCCACGAGAAGGAT	ATATGTACGACGGGCATG	191743
*skn-1*	CTGGCATCCTCTACCACCAC	TTGGTGATGATGGCCGTGTT	177343
*atf-7*	CGGAACAATGTCGGATCTTT	CATGTCGAGTACCGCGTTTA	175587
*clec-60*	CGGTTTCAATGCGGTATGGC	TGAAGCTGTGGTTGAGGCAT	191384
*clec-85*	CCAATGGGATGACGGAACCA	CTTCTGTCCAGCCAACGTCT	177068
*abf-2*	CCGTTCCCTTTTCCTTGCAC	GACGACCGCTTCGTTTCTTG	266826
*lys-1*	GTACAGCGGTGGAGTCACTG	GCCTTGAGCACATTTCCAGC	179428

### Western blotting analysis

For cells, THP-1 cells were first differentiated into macrophages with 100 ng/mL PMA and then treated with log-phase MRAB 0227 cells (1 × 10^7^ CFU/well) for 4 h. After washing two times with PBS to remove the bacteria in solution, the cells were incubated with the peptides 12.5 μM 2K2L and 6.25 μM 2K4L for 1 h, 2 h and 4 h, respectively. The cells were collected by centrifugation at 1000 rpm, resuspended in RIPA lysis buffer with a mixture of protease inhibitors and phosphatase inhibitors and lysed on ice for 30 min. The lysates were centrifuged at 12,000 rpm for 10 min at 4 °C, and the protein extractions were obtained. PMB (1.56 μM) was used as a positive control. For nematodes, age-synchronized L4 worms were infected with log-phase MRAB 0227 or AB 22933 for 24 h. After the surface-colonized bacteria were removed with M9 buffer, the nematodes were transferred to new NGM plates and treated with the peptides for 6 h. For LPS*-*induced septic mice, mouse liver tissues were fully ground with an automatic sample fast grinder and then suspended in RIPA lysis buffer. The protein extractions were obtained by centrifugation of the supernatant for 10 min at 12,000 rpm at 4 °C.

The concentrations of protein from cells, nematodes and mouse liver tissues were quantified using a bicinchoninic acid (BCA) Protein Assay Kit (Beyotime, Shanghai, China). The proteins were separated on a 10% SDS polyacrylamide gel and then transferred to a PVDF membrane. PVDF membranes were blocked in TBST containing 5% skim milk at room temperature for 2 h and incubated with primary antibodies overnight at 4 °C. After washing with TBST, the membranes were incubated with a horseradish peroxidase-conjugated anti-rabbit IgG secondary antibody (1:5000) for 2 h at room temperature. Finally, the immunoreactivity of the membranes was developed using an ECL chemiluminescence substrate (Thermo Scientific, USA). Protein-antibody complexes were detected using an Azure c500 imaging system (Azure Biosystems, USA). Band intensities were measured using ImageJ software.

### Mice and ethical statement

SPF C57BL/6 male mice (6–8 weeks, 18–22 g) were purchased from Liaoning Changsheng Biotechnology Co., Ltd. and housed under standard conditions of light and temperature in the Laboratory Animal Center at Liaoning Normal University. Mice had free access to standard laboratory chow and water. All animal experiments in this study were approved by the Animal Use and Care Committee of Liaoning Normal University (the ethical approval number: LL2023063).

### LPS-induced shock mouse model

Seventy-five male C57BL/6 mice were randomly divided into five groups (n = 15/group): (1) 0.9% NaCl (control group); (2) LPS (15 mg/kg weight); (3) LPS + dexamethasone (DXM, 5 mg/kg weight); and (4)–(5) LPS + 2K4L (0.5 and 1 mg/kg weight, respectively). The LPS-induced shock mouse models were established as follows: briefly, mice were injected intraperitoneally with 15 mg/kg *E. coli* 0111:B4 LPS (Sigma‒Aldrich, ≥ 500,000 EU/mg). After 24 h of LPS injection, 0.9% NaCl or 2K4L at different concentrations or DXM (dexamethasone) was injected i.p. into the mice for 7 days. Status and weight were regularly monitored every day. The survival rates of mice within 7 days were calculated. At Days 2 and 7 of peptide administration, mice were sacrificed, blood samples were collected for later analysis of liver function and inflammatory factor release, and lung, kidney and liver tissue were collected for histochemical observation and western blotting.

### Liver function assay

The activities of ALT and AST in mouse serum were determined to examine the magnitude of hepatic injury by using a VITROS 5600 automatic biochemical immunoassay analyzer (Ortho-Clinical Diagnostics, Inc.).

### Histological observation by H&E

At 2 and 7 days of peptide treatment, mouse livers, lungs and kidneys were collected and rinsed three times with normal saline and then immediately fixed in 4% formaldehyde before being embedded in paraffin. Paraffins were sliced and stained with hematoxylin and eosin (H&E). The pathological changes in mouse tissue sections were observed under a microscope and photographed.

### Immunohistochemical analysis

Liver, lung and kidney tissues were incubated in 4% paraformaldehyde solution overnight, dehydrated, and embedded in paraffin. Paraffin-embedded samples were cut into 5 to 7 μm thick sections and mounted onto poly-l-lysine-coated glass slides. After deparaffinization and hydration, sections were incubated in 5% normal goat serum for 30 min. Antibodies against phosphorylated p38 (*p*-p38), ERK (*p*-ERK), JNK (*p*-JNK), IκB (*p*-IκB) and NF-κB p65 (*p*-NF-κB p65) were added to the tissues, which were covered with plastic wrap overnight at 4 °C. After incubation with secondary antibodies for 30 min, the samples were incubated with HRP for 15 min with a DAB Horseradish Peroxidase Color Development Kit. The samples were visualized using 3,3′-diaminobenzidine (DAB) for 5 min protected from light and counterstained with hematoxylin.

### Statistical analysis

All experiments were performed in triplicate. The results are generally expressed as the means and standard errors. The paired Student’s t-test was used to test for statistical significance. Significance is indicated as *for p < 0.05 and **for p < 0.01.

### Supplementary Information

Supplementary Information.

## Data Availability

The datasets used and/or analysed during the current study available from the corresponding author on reasonable request.
