# Supplementary material for: Antimicrobial peptide 2K4L inhibits the inflammatory response in macrophages and Caenorhabditis elegans and protects against LPS-induced septic shock in mice
Source: Sci Rep. 2024 Jul 2;14:15093. doi: 10.1038/s41598-024-64511-9 (PMC11219918; doi:10.1038/s41598-024-64511-9)

Mice

2 天 GAPDH

2 天 ERK

2 天 p-ERK

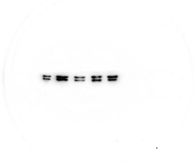

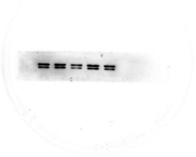

2 天 P38

2 天 p-P38

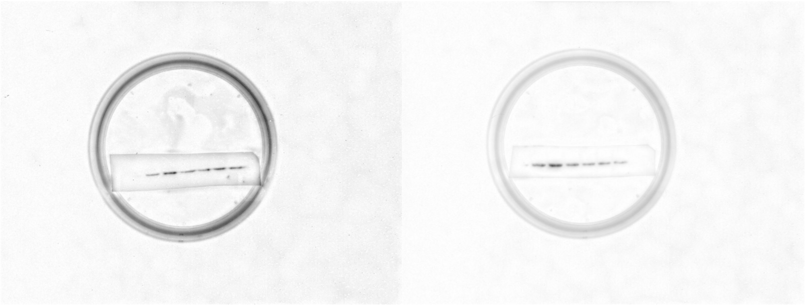

2天 JNK

2天 p-JNK

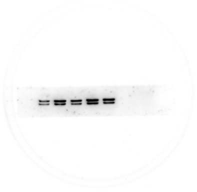

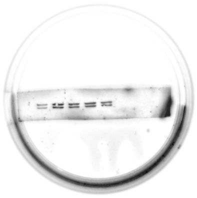

2天 IkB

2天 p-IkB

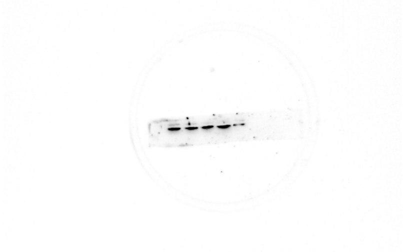

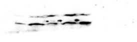

2 天 NF-kB

2 天 p-NF-kB

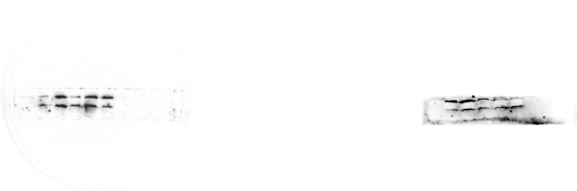

7 天 GAPDH

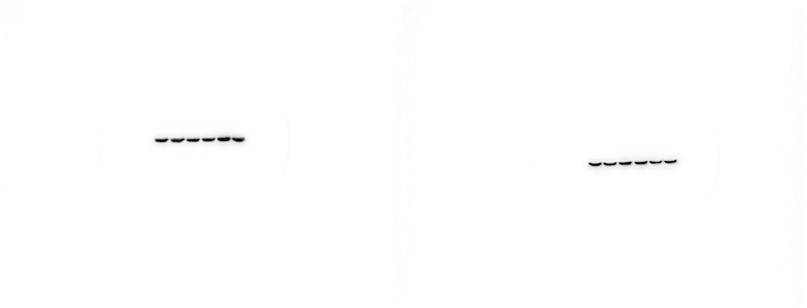

7 天 ERK

7 天 p-ERK

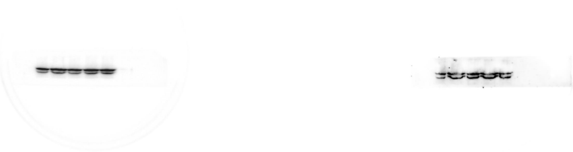

7 天 P38

7 天 p-P38

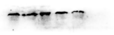

7 天 JNK

7 天 p-JNK

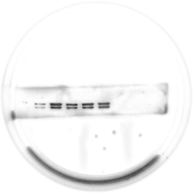

7 天 IkB

7 天 p-IkB

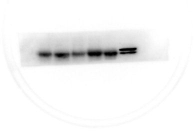

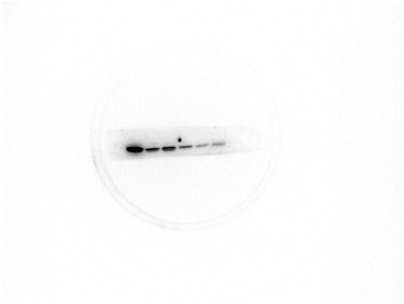

7 天 NF-kB

7 天 p-NF-kB

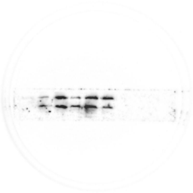

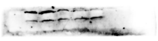

C. elegans

β-actin

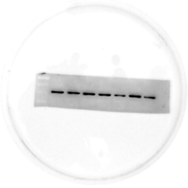

P38

p-p38

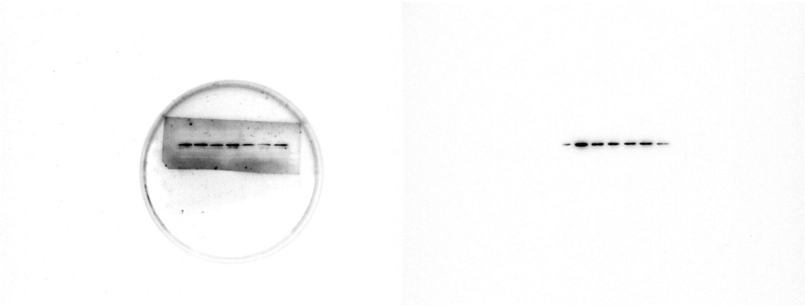

THP-1

GAPDH

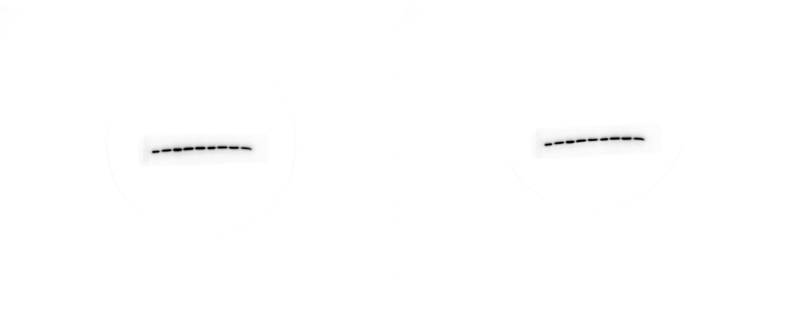

Ikb

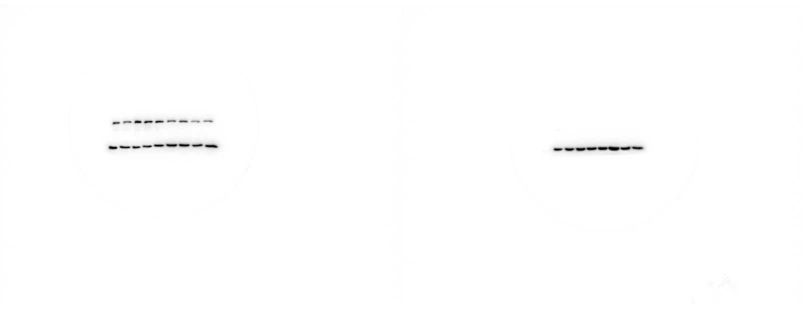

p-Ikb

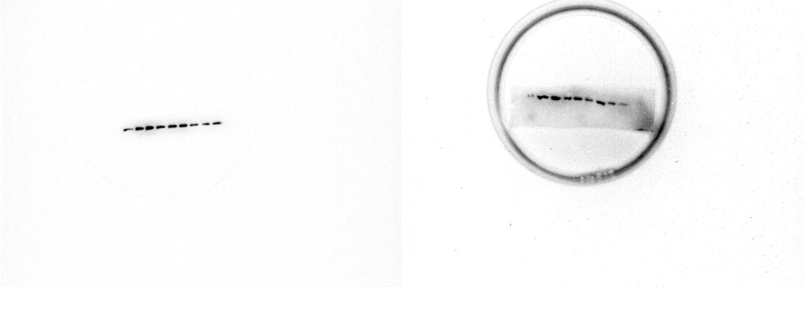

NF-kB

p-NF-kB

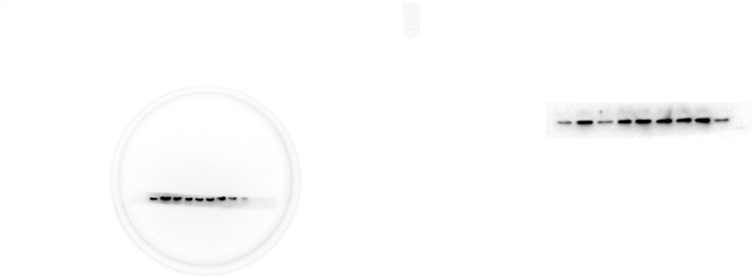

Supplement: Supplementary file 1 — Supplementary Information. [file 41598_2024_64511_MOESM1_ESM.pdf]
